# A critical qualitative study to understand current black women medical student perspectives on anti-racist reform in US medical education

**DOI:** 10.1080/10872981.2024.2393436

**Published:** 2024-08-20

**Authors:** Nouran Ghanem, Debora Goetz Goldberg, Eldesia Granger, Jennifer R. Warren, Gilbert Gimm

**Affiliations:** a College of Public Health, Department of Health Administration and Policy, George Mason University, Fairfax, VA, USA; b Department of Health Administration and Policy, College of Public Health, and Affiliate Faculty, Center for Evidenced-Based Behavioral Health, Department of Psychology, George Mason University, Fairfax, VA, USA; c Private Practice Physician, Alexandria, VA, USA; d Institute for Health Equity Education & Research, Atlantic City, NJ, USA; e Department of Health Administration and Policy, College of Public Health, George Mason University, Fairfax, VA, USA

**Keywords:** Medical education, curricular reforms, racism, anti-racism, black women medical students, qualitative research, critical qualitative inquiry

## Abstract

**Purpose:**

The US medical education system has a long-standing history of omitting evidence and perpetuating false pseudo-scientific beliefs on the complex and nuanced relationships between race, racism, and health disparities. There is an urgent need to identify and address the historical influence of systemic racism on the current curriculum, organization, and culture of US medical education. The goal of this study was to understand Black women medical student perspectives on race and racism in current medical school training and their recommendations to inform anti-racist action in US medical education.

**Method:**

The authors conducted a critical qualitative study to understand the perspectives of Black women medical students on issues surrounding race and racism in relation to US medical education. To their knowledge, this is the first study to use qualitative research methods to understand current thinking on the need for anti-racist pedagogy in medical school education among Black women medical students in the US.

**Results:**

The interviews revealed critical limitations in the teaching of race, racism, and racial disparities, including a lack of historical depth, continuity, and evaluation of this content; lack of actionable guidance to address racial disparities in clinical practice; and dissonance between emerging anti-racist content and national licensing examinations. The qualitative data yielded several anti-racist strategies and practices that can be implemented in US medical schools to redress historical curriculum limitations and better prepare future generations of physicians to care for marginalized populations.

**Conclusions:**

This study provides actionable feedback on needed reforms to redress US medical school curriculum limitations as it relates to race, racism, and racial disparities.

## Introduction

The social uprising of 2020 in response to high-profile police killings brought into motion public calls to confront and address our nation’s historical legacy of slavery and systemic racism. This legacy is especially apparent in the US health-care system where inequitable access to quality care and poor health outcomes persist among minoritized and marginalized communities – a reality brought into sharp focus by the COVID-19 pandemic [1]. While the medical community has embraced an expanded framework on the social contexts influencing health (i.e., the social determinants of health), it has been slower to explicitly acknowledge and address systemic and structural racism as a cause of health disparities. This gap in awareness and knowledge creates a need to critically examine how systemic and structural racisms in medical education is taught, discussed, and interrupted.

The evidence on the impact of racism on health outcomes is significant enough to recognize it as a determinant of health. There are consistent associations between: (1) interpersonal discrimination and poor mental and physical outcomes; (2) chronic stress as a result of exposures to racism and premature biological aging (known as ‘weathering’); and (3) the effects of structural racism (e.g., racial residential segregation) and adverse health outcomes [2–4]. Research on racial disparities reveals that even after accounting for socioeconomic and sociodemographic variables such as income and education, racial disparities continue, suggesting that systemic racism independently contributes to unequal health outcomes in racialized communities [5]. However, the historical context underpinning systemic racism and its effects on health are not a required component of medical curricula.

US medicine has a history of reifying race as a biological construct [6] despite broad scientific consensus that there is no genetic basis for race as a biological category and biological differences between racial groups do not exist [6–10]. Rather, the social process of racialization (i.e., assigning social meaning to racial categories) and racism (i.e., a system of advantage based on race) define race as a social construct [7–10]. The historical process of racialization can be traced to the legacy of slavery and eugenics movements that contributed to negative conceptions of the Black population as susceptible to illness, laziness, pain insensitivity, and beliefs that endure today [2,11]. Medical education plays a role in preserving the legacy of racism by conflating race as a ‘risk factor’ to pathology, a harmful conflation that manifests itself in individual clinician biases and care behavior and commonly used race-adjusted clinical algorithms, the effects of which ultimately lead to the disparate treatment of and medical mistrust among Black patients [12–14].

There has been a long-standing conversation to integrate concepts of racial equity in US medical training, leading to a number of state-level efforts to require racial equity and implicit bias training among clinicians [15–19]. The urgency for this work increased in the wake of recent events, giving rise to additional efforts, initiatives within the medical school setting to examine and teach about the role of racism in the health outcomes and care of racialized populations; and race-based practices in perpetuating racist beliefs, attitudes, and behaviors in training medical students [20–35]. This is a promising step forward given that educational interventions may improve clinical empathy in physicians and reduce bias against patients of color [18]. However, to date, few standards exist to guide these efforts and the sensitive nature of these efforts requires ‘meeting educators and students where they are’ on race-related matters [6,18]. We further find that emerging curriculum reform efforts do not specifically respond to, center, and incorporate the experiences of Black women medical students who are often excluded from anti-racist discourse [36].

Sharp and Hixson et al. [36] performed a recent narrative review concluding that there is negligible empirical research centering on the perspectives of Black women learners in medical education. Black women have multiple marginalized identities and a nuanced experience of gendered racism, being ‘behind’ both Black men and White women – an experience not accounted for in medical education literature [36]. We affirm that a focus on their voices is necessary to inform national anti-racist discourse and to ensure that anti-racist reforms in medical education are sustained, relevant, and sensitive to populations enduring systemic harm. This forms the basis for a gap in the literature this study seeks to address.

Centering the Black women medical student voice is necessary to aid in transforming traditional knowledge production in medical disciplines as previous literature by White scholars may downplay the ‘systemic depth’ of racism [37]. This study overcomes this effect by drawing upon the principles of critical qualitative inquiry to center the voices we seek to serve with this new knowledge. The goal of this study is to understand Black women medical student perspectives on race, racism, and anti-racism^1^ in current medical school curricula. To our knowledge, there exists no qualitative investigation which centers the voices of Black women medical students to inform anti-racist actions in US medical pedagogy and praxis.

## Materials and methods

### Study design

This research has been reviewed and approved by the George Mason University Office of Research Integrity and Assurance on 17 August 2023. We conducted virtual one-on-one semi-structured interviews with current medical students identifying as Black or African American women in the US The virtual format was used to increase the convenience to participate given the many competing demands on the time of medical students. In addition, it served to increase the geographic representation of participants. Informed consent took place prior to the interviews. Participants who completed the interview received a monetary incentive of $100 as compensation for their time.

The methodological orientation that underpins the design is a critical qualitative inquiry. Specifically, the semi-structured questions and analytical approach were influenced by qualitative researchers, Norman Denzin and Yvonne Lincoln, who call on researchers to use qualitative inquiry to meet a social justice need and not just produce new knowledge [38,39]. Doing so means moving from the traditional paradigm of qualitative research as a tool to merely observe and interpret the world towards an instrument to change it – to address the ‘forms of inequality and discrimination that operate in daily life’ [38]. According to Denzin and Lincoln, this paradigmatic shift is operationalized by (a) ‘giving precedence … to the voices of the least advantaged groups in society’, and (b) using the qualitative inquiry [to] elicit change in people and policy.

### Participant selection

We used purposeful sampling, including convenience and ‘chain referral’ (snowball) sampling [40], to recruit medical students identifying as Black or African American women from medical schools across the US. Purposeful sampling was used to increase the chances of reaching a sample size necessary for ‘saturation’, given the small demographic makeup of matriculants into medical school identifying as Black or African American, which was 10% in 2022–2023, a number that has overall only marginally changed overtime [41]. Black or African women made up 8.2% of the matriculants. This number is even smaller when compared to the total enrollment population, in which Black or African American women make up around 4.5% from 2020- 2022 [36]. Further, Black women physicians only make up 2% of the active physician workforce [37]. This sampling strategy was initiated through email outreach to members of the Student National Medical Association, an affinity organization for current and future underrepresented minority medical students dedicated to supporting these students and increasing the number of ‘clinically excellent, culturally competent and socially conscious physicians’ [42]. This outreach led to the first eligible participant. The nature of the snowball sampling was explained at the conclusion of the interview with her, after which she shared the recruitment announcement with a medical student peer.” This process continued until saturation was reached. The total sample size was 30, inclusive of first year (M1), second year (M2), and third year (M3) students. Participants represented schools from across the east coast, west coast, mid-west, and southeastern regions of the country. The states or territories of the schools represented included Alabama, Arizona, California, Illinois, Indiana, Louisiana, Massachusetts, New Jersey, New York, North Carolina, Pennsylvania, Texas, and Washington DC The schools represented also spanned different funding statuses and levels of national prestige, including public, private-funded schools, Historically Black Colleges and University (HBCUs), and Ivy League institutions.

### Data collection

In-depth, semi-structured one-on-one virtual interviews of 60-minute duration, using Microsoft Teams, were conducted. To collect sociodemographic data on the participants, an online pre-interview questionnaire was developed using Qualtrics and distributed via email prior to the interview.

The lead author (NG) facilitated the interviews and conducted the analysis; she identifies as a woman of color of North African descent. An interview guide was used consisting of semi-structured questions on the following topics: understanding and perception of the legacy of racism in US medicine; perceived limitations, and omissions of current medical school curricula with regard to the teaching of race-based health inequity and disparities; experience and perspectives about anti-racist curricula efforts and best practices or limitations related to such efforts; the general student experience, and recommendations to introduce anti-racist practices in US medical school education. To increase the validity of the data collection and analysis process, the interview guide was reviewed by two researcher team members (EG and JW), a physician and a subject matter expert, respectively, who both identify as a Black woman, in addition to two other research team members with expertise in qualitative research. See ‘Supplemental Digital Appendix 1: Semi-structured Interview Questions’ for the list of questions. The interview guide was ‘piloted’ with the first two interviewees by asking the following questions at the conclusion of the interview: ‘Was there anything that could have been asked or worded differently; and was there anything that could have made the process more comfortable for you?’. Recruitment was concluded once ‘saturation’ 36 was achieved (i.e., significant reoccurrence in concepts, arguments, and sentiments captured).

### Data analysis

The interviews were audio-recorded, transcribed verbatim, and de-identified for qualitative analysis using NVivo version 12 (QSR International). Participants were notified at this stage and informed that they may request the transcript to review it for accuracy and retract or edit anything, to ensure their comfort with the transcript prior to analysis. The analysis consisted of three phases, drawing on best practices in coding textual data developed by Saldana and Wolcott [43,44]. The first phase involved reviewing each interview transcript twice to notate the presence and frequency of significant concepts and define an initial set of codes. The second phase was conducted to further refine the codes in developing the codebook and coding structure (e.g., combining, adding, or removing codes). The third and final phases were performed to transform the coded data into key themes. In alignment with Denzin [38] and Lincoln’s [39] qualitative framework, the analysis focused on actionable feedback for the medical community to act on to advance an anti-racist lens in medical education.

Two validity measures were employed to increase the credibility of the data. The first was ‘member checking’ [45] to validate the interpretations of the data – we presented thematic descriptions in written format to a subset of participants and solicited written or verbal feedback to check on accuracy and resonance of the interpretations. Second, EG and JW, who both identified as Black women, reviewed the thematic descriptions to identify nuances, potential mischaracterizations, or needed clarifications of the data. Both the subset of participants who engaged in ‘member checking’ and the review by the two members on the research team aided in validating the interpretations of the data, to ensure an accurate reflection of what was shared.

## Results

All 30 participants identified as Black or African American women with the majority (n = 20; 66%) identifying as either a first-generation American immigrant of African, Haitian, Caribbean descent (i.e., a person who has arrived in the US from an African country, Haiti, or the Caribbean), or a second-generation American of African, Haitian, or Caribbean descent (i.e., a person who was born to first-generation immigrants of this background). A minority of the participants (n = 10; 33%) identified as a Black or African American individual with generational roots in the US. The majority of the participants were in their second year of medical school (n = 27; 90%). Slightly over half of the participants are between the age of 25–34 years of age (n = 17; 57%) with the other half consisting of participants between 18 and 24 years of age (n = 13; 42%). The majority of students are not first-generation college graduates (n = 19; 63%), but first in their families to pursue a terminal degree (n = 25; 83%). Lastly, the majority of participants are using loans to pay for medical school (n = 23; 76%). Table 1 below presents the sample characteristics.

**Table 1.** Participant sociodemographic characteristics.

Sociodemographic characteristic	n (%)
**Generational identity**	
First-generation African, Haitian, Caribbean descent	*n*= 8 (26%)
Second-generation African, Haitian, Caribbean descent	*n*=12 (40%)
Generational roots in the US	*n*=10 (33%)
**Gender identity**	
Woman	*n*=30 (100%)
**Age range**	
18-24	*n* = 13 (*n*=43%)
25-34	*n* = 17 (57%)
**Educational history**	
First-generation college graduate	*n*=11 (35%)
First-generation terminal degree student	*n* = 25 (83%)
**Loan assistance**	*n* = 23 (76%)

The interviews broadly revealed that the scope, frequency, format, and the degree to which race-related content is integrated across the four-year experience varies widely across schools. These findings also demonstrate that anti-racist efforts are largely championed by the students themselves, creating challenges related to faculty buy-in and the risk of the ‘minority tax’ effect, whereby the burden to advocate on issues of racial justice is disproportionately shouldered by people of color, drawing time away from coursework and exams.

Below, we present the domains and key themes from the interviews. These themes relate to (1) limitations in the current curriculum, (2) strategies to advance anti-racist curriculum reform, and (3) barriers to scaling curricular reform at a national level. Please see ‘Supplemental Appendix 2: Key Theme Participant Quotes Table’ for a table of illustrative participant quotes by key theme.

### Critical limitations on course content related to race and racism

Overwhelmingly, participants across the represented schools described major perceived weaknesses and issues they would like to see addressed in future efforts to revise the curriculum. The key themes from commonly cited weaknesses are summarized as follows.

### Lack of depth and historical lens related to racism and racial health disparities

Nearly all participants relayed that the legacy of slavery is never mentioned in discussions related to racial health disparities, and the historical nature of racism in the US in relation to medicine is not explained. Instead, students reported that current health disparity content is focused on the disparities within the surrounding community of the medical school and the relationship between the community and the institution; present-day disparities along racial and non-racial dimensions, e.g., gender identity, sexual orientation, immigrant status, etc.; and the way in which social factors affect disparities, e.g., lack of access to care, healthy food, etc. The omission of the historical context of slavery and its relationship to present-day disparities was rationalized by students with comments that it may be ‘assumed’ knowledge or omitted due to the perception that it may be too sensitive or upsetting to their White counterparts.

They conveyed that this omission does a disservice to future physicians as it reinforces ambiguity around the relationship between race and disparities. For example, some participants expressed concern that course content emphasizes that Black communities are disproportionately affected by certain diseases and conditions due to their race and not due to systemic racism.

I don’t think that I’ve seen anything specifically talk about slavery and the impact there’s always, you know, courses, where we cover the impact of socioeconomic status, but no one is actually talking about why those socioeconomic differences exist in the first place. They’re just going to say they exist, and you should keep that into account in your future practices as a doctor …

But if you don’t have the historical context, it just seems like we all woke up and somehow Black people aren’t healthy in America?…

Participants stated that this curriculum limitation reinforces a complacency with the status quo of disparities and creates confusion around why a focus on emphasizing and addressing anti-Black racism (as opposed to racism across all racial groups) is necessary to dismantling problems of racism and inequity in health care.

### Lack of nuanced explanation and exploration of racial inaccuracies, stereotypes, and ongoing misuses of race in medicine

Many participants expressed that there are some instances in which a lecturer may indicate that a certain clinical guideline or diagnostic tool is out of date due to their misuse of race; however, they relayed that the lecturer will not explain the false assumptions associated with race that made it inappropriate for continued use nor explain an appropriate alternative. One participant described this lack of explanation as problematic since the logic behind race-based assumptions could help in updating clinical diagnostic methods. Several participants also noted that race is not explicitly described as a ‘social construct’ and that there are certain racial disparities that are not explained or disentangled in a way that clarifies race as a social construct. For example, one participant described how type 2 diabetes is so strongly associated with being Black in their observation of the course content that they are concerned future physicians will overlook the possibility of type 1 diabetes in Black or African Americans.

I’m doing type one diabetes research right now, and I hear so much Black people have been misdiagnosed because they come in and they’re just like, oh, you got type 2 diabetes … . But I have yet to see in my curriculum – make sure that this person doesn’t have type one diabetes.

Participants noted that the constant presentation of these chronic conditions as disproportionately affecting Black populations creates the assumption that Black people are genetically predisposed to certain diseases and conditions; however, they are uniquely aware of the systemic factors that lead to the poor health outcomes in Black and other racialized communities.

Lastly, participants commented on how they commonly hear stereotypes about the Black body from their classmates that are not collectively addressed anywhere in the curriculum – the beliefs that Black people have ‘thicker skin’ and greater pain tolerance were examples of comments heard in the classroom by students.

## Lack of cohesion and continuity of topics introduced

Nearly all participants indicated that in addition to the lack of historical context in relation to disparities, the content related to health disparities consists of many disparate topics such as social determinants of health, medical ethics, health among marginalized communities, etc. This was communicated by participants as ‘scattered,’ rather than a cohesive, integrated part of the curriculum. Participants elaborated that the ‘scattered’ approach undermines learning of the content in a meaningful and iterative way, i.e., building upon fundamentals to more complex topics.

I couldn’t even tell you the last time we had a meeting regarding race because it’s just sprinkled here and there. It’s sporadic.

Similarly, several participants noted that this content is usually included after basic science content or after a long day of lectures or exams, which comes across as an ‘afterthought,’ or fragmented, undermining student engagement due to potential exhaustion from mentally taxing tasks. Finally, most participants relayed that in relation to the frequency of basic science coursework and clinical training, content on race and racial disparities represents a small fraction of the overall curriculum and is presented only in the first year or two, leading many participants to note that it is ‘surface-level,’ insufficient, and lacks continuity across the four-year experience.

## Lack of actionable guidance and application of knowledge in the context of clinical practice

Many participants relayed that in instances in which disparities are discussed, it often creates discomfort among students who identify as Black or African American due to the repetitive nature of disparities communicated about this population and the way in which this information is delivered. Participants described their discomfort with discussion of certain conditions such as HIV, sickle cell disease, type 2 diabetes, and maternal mortality. When reflecting this sentiment, many participants conveyed that they internally asked what can be done about these disparities. They expressed that it feels like ‘statistics are thrown at them,’ but no one is explaining how to translate that knowledge into action in the context of clinical practice.

You just get all these grim statistics, and you’re like, wow, I’m more likely to die if I have a kid. Like what am I supposed to do with that information? And even not necessarily for myself but like for my patients. What am I supposed to do with that information? So maybe actionable steps instead of just learning these statistics.

By only limiting the content to the problems, students walk away with a feeling of helplessness but convey an eagerness to use their position and privilege as future physicians to affect change and to serve the communities they come from.

## Lack of qualified academic instructors trained on race and racism in medicine

As alluded to above, many participants indicated that disparities affecting Black populations can be presented by professors/lecturer (s) in an insensitive way that makes students who identify as Black or African American feel out-of-place and alone as those disparities are ‘close to home’ but are taught in ways that otherize that population as sicker and lesser. Who should ideally teach this content emerged as another theme – many participants relayed a two-part comment on the background of individuals best suited to provide instruction on disparities and other anti-racist topics. First, many participants noted that they would be more comfortable seeing an individual who identifies as Black or African American teaching this content, but noted several nuances to this suggestion, as follows:

- More than their racial identity, the instructor(s) should be trained, equipped, and educated about issues of race and racism in medicine and ideally have a track record of working with underserved communities. As long as they have a demonstrated background related to anti-racism, anyone who has the passion for this work should be a candidate. Limiting recruitment to a specific racial group may create the impression that this work is not everyone’s work to do, in addition to the risk of a ‘minority tax’ effect.
- Relatedly, these educators do not have to hold an MD credential. Individuals with disciplinary backgrounds from the social sciences (e.g., sociology and psychology) and the humanities (e.g., critical theory), may bring more valuable expertise in teaching this content than those with a clinical background alone.

I know that there are thought leaders in these areas that exist on all of these campuses. So, for me, it’s kind of frustrating that we have people who are doing research and the work … Why are we not bringing in these people to provide that education?

## Dissonance between curricula changes and national board and licensure exams

Many participants indicated that while their respective curriculum has begun to introduce content on racial disparities and systemic racism, the major notion underlying this content – race is socially constructed – starkly conflicts with the racialized prompts still present on the major board and licensure exams, i.e., the United States Medical Licensing Examination (USMLE) standardized exams, critical to becoming a practicing, licensed physician in the US. Students elaborated that in the classroom setting, they are gradually being taught to disentangle race with racial disparities and to instead look at the structural factors underlying prevalence of certain pathology; however, they are simultaneously trained to recognize prompts with an expected, single answer pointing to a racial group.

As far as national board, that is something that will come up, there will descriptors, and you’re immediately supposed to think of whatever problem, right? Just because it says Black 50-year-old man …

This experience was described as a simultaneous ‘learning and unlearning’ which students find conflicting and confusing. Several participants noted that given the lag in change in the design of standardized exams, a minimum requirement among faculty should be to caveat and delineate instances when content is geared toward performance on these standardized exams versus what should be understood in real-world practice as future physicians.

### Strategies for anti-racist curriculum reform

A number of major suggestions were reoccurring across the interviews, namely the need to: (1) Hold students accountable for processing the content in a similar manner as the required basic science, pre-clinical content, (2) Engage with the content in a deeper manner, to ‘critically reflect’ on issues surrounding racism in medicine, given the stakes of exhibiting implicit and explicit biases in clinical practice, (3) Use a small-group learning format rather than a didactic, mass lecture format due to the sensitive nature of these topics, and (4) Engage and interact with racialized and marginalized communities to expose students from more privileged backgrounds to the lived experience of these communities. These suggestions are summarized below.

## Evaluate anti-racist content on the same standard as basic science coursework

Many participants noted that the mental load associated with the basic science content and the major standardized exams is significant and can be overwhelming; therefore, any content that is treated as optional, or not accounted for in course grades, exams, etc., is likely to be dismissed so that students can narrow their focus to what is necessary for performing well. This pushes anti-racist content to a lower level of significance in a student’s decision criteria.

There was an option … I think it would be the only anti-Blackness and racism lecture last year, which was done as a pilot for, and it was optional for students, basically no students showed up and the students who did show up were Black.

Creating an accountability mechanism through evaluation would elevate its significance, some participants suggested ensuring that there are graded assignments or questions on exams. Many participants did not advocate for a traditional test approach to evaluate this content as it requires critical reflection and interrogation of one's own biases, assumptions, and preconceived notions surrounding race.

## Provide students with opportunities to critically reflect on biases at an individual and broader level

Participants relayed that the current content in this area is ‘surface-level’ and does not allow students the ‘space’ to critically reflect on their own biases and how racism manifests itself in the health system. To improve this, they offered suggestions to create more space for critical reflection. One suggestion was to introduce students to a panel of experts from multiple disciplines within and outside the institution who can speak on these issues and engage students in interactive discussions. Another suggestion was to invite patients from marginalized backgrounds to come speak about their lived experience with barriers they encountered in their care experiences and the negative impacts of biases they may have been affected by.

Or even bringing in people that actually are affected by stuff like that and just discussing … I have a friend who has sickle cell and she has to go through loopholes just to get certain pain medications because she fits the demographic that could abuse these medications. You know, she has to fight with physicians just to be treated equally. So someone like that coming to talk about the issues that they face

This exposure would lend itself to discussions on how to interrupt one’s own biases and respond to the witnessing of implicit and explicit bias in the clinical setting. Some participants indicated that listening and reflecting on the stories of real patients would be a lot more powerful than taking the implicit bias association test (IAT), which they commented did not lead to meaningful engagement or discussion on how to address one’s biases.

## Shift away from the traditional lecture format to in-person small-group sessions and discussions

Participants unanimously conveyed that the traditional didactic lecture format is not conducive to teaching content on race and racism and that these topics deserve to be treated with more care and sensitivity. Towards that end, they suggested that the format for a specific course focused on these topics, or a course component within other courses, should entail a teaching method to ensure students acquire a ‘baseline’ understanding around a topic (e.g., required reading before class or an initial lecture), followed by an in-person small-group session to enable students to discuss complex questions amongst themselves without fear of judgement from a large audience. Many participants indicated that their school already has a small-group format within the current curriculum structure so using it towards this end would be a minimal change.

I think I probably get a little bit more out of small groups than necessarily lecture style because I think questions tend to generate conversations on the topics and that resonates more with me rather than being lectured at.

## Integrate opportunities to interact and engage with marginalized communities

Many participants indicated that learning about racism-related topics cannot be limited to the classroom setting and that engaging and interacting with individuals from marginalized and under-resourced communities within parts of their respective school’s state is key to allowing students the opportunity to understand and interrogate their discomfort with certain communities so that when they become physicians they do not act on the basis of their biases and discomfort with people who are unlike them.

… People discuss these issues in the classroom, but then it’s then you leave the classroom and never really interact with anybody who’s different from you or comes from a different background from you. So, I think maybe just more community engagement.

Participants stressed that this is particularly important given that the student population in medical schools is comprised of individuals from more resourced backgrounds and less diverse communities. A few participants cautioned that this work must be done with care and cultural humility to ensure that undue burden is not placed on these communities for teaching opportunities.

## Learn from emerging best practices to move towards an anti-racist curriculum

Several participants described what they considered best practices in relation to revising norms and practices within medical schools to equip students to care for diverse populations. Two major best practices were reoccurring. The first was for the pathology course faculty to be trained in historical problems with race-based medicine. Students emphasized that this faculty member has the opportunity to continually stress and correct inappropriate assumptions surrounding race, genetics, and racial disparities. The second practice was to move from race descriptors in the clinical narrative to ‘person-first’ language throughout course material and medical record documentation. This means the description of the patient consists of their age, symptoms, clinical history, and sociodemographic characteristics but not the patient’s racial identity. This also means transitioning from using descriptors that characterize someone’s identity on the basis of a presenting condition, e.g., not calling someone an alcoholic but someone who suffers from alcohol use disorder.

### Obstacles to scaling anti-racist curriculum reforms at a national-level

Overwhelmingly, participants conveyed they anticipated various forms of resistance that would make progress long and slow. These limitations are four-fold. The first is resistance from the medical school administration. Participants noted that current efforts to improve content and delivery of race, racism, and disparities-related material were met with a lack of support from administrators and influential faculty. The second is an incongruence between the medical school classroom curriculum and the affiliated academic Medical Center environment where changes are not always ‘carried over’ to the clinical training portion, e.g., using ‘person-first’ language in the classroom but not in the clerkship. The third is the politicization of race at the state and federal level in which policymakers and elected officials have acted to censure or undermine the inclusion of these topics. The June 2023 Supreme Court ruling on affirmative action was cited by the students as an example of the unfavorable political environment for anti-racist change with participants relaying that it has already produced visible effects on the diversity of the medical school student body. Participants noted that the long-term implication of decreased diversity is less diverse people at the leadership level with decision-making authority to influence curriculum content, which would serve to preserve the status quo. More than one participant noted that a mitigating factor to these obstacles would be that accrediting bodies setting standards for educational content and requirements, including the Liaison Committee on Medical Education (LCME) for medical education quality standards, and the USMLE for standardized examination, create new anti-racist curricular requirements and changes that would be mandatory for medical schools nationally to adapt to and integrate.

Please see ‘Supplemental Appendix Table S2: Key Theme Participant Quotes Table’ for representative quotes on obstacles to scaling reforms.

## Discussion

Medical schools are responsible for preparing future generations of physicians to meet the nation’s care needs and play a critical role in the functioning of the US health system. The results of this study illuminated several key challenges and opportunities for medical schools to address in the process of evaluating existing and creating new curriculum content on issues concerning race, racism, and racial health disparities. Significant gaps were articulated regarding the historical contextualization of race and racism in the US; lack of nuanced explorations of racial inaccuracies and stereotypes; continuity of this material throughout training; and access to faculty equipped to these topics. Overall, participants voiced several obstacles to scaling changes in medical schools and at the national level, including potential attitudes of medical school administration/leadership and the broader political climate. Notable strategies in moving toward an anti-racist curriculum approach were affording time to reflect on individual biases and holding students accountable for retaining and processing content.

The study results call for concerted action within the medical school community to reform medical school training, to ensure that it comprehensively and critically addresses the relationship between race, racism, and health disparities. The perspectives centered in this study inform the following reforms and strategies that medical schools can independently and immediately act upon:

1. Reviewing current curriculum content on health inequity and racial disparities and moving towards integrating required content on race, racism, and racial disparities with a historical lens in a longitudinal, cohesive way throughout the curriculum that is on par with the quality standard of basic science coursework.
2. Facilitating in-depth exploration of assumptions and beliefs surrounding race and racial disparities (e.g., unpacking beliefs surrounding pain tolerance of Black individuals) and providing students with exercises to critically reflect on their own biases and discomfort with racial differences and racialized realities.
3. Creating productive opportunities for students to engage with marginalized communities throughout the 4-year experience in a way that is informed by methods and practices that help ensure respect for and empowerment of these communities.
4. Aligning changes in the classroom curriculum to the clinical training within the affiliated Medical Center using a change management approach, e.g., identifying a ‘change champion’.

This study builds on the ongoing discussions and efforts to train practicing and future physicians on racial equity and related concepts. Several of the thematic findings and recommendations align with the growing literature on the need to introduce medical students and trainees to the historical legacy of racism and its influence in medicine, including the recommendations of Blanchard et al., to move beyond ‘cultural competency’ towards ‘structural competency’ (i.e., the understanding of how upstream and structural factors impact downstream health outcomes); integrate implicit bias mitigation training; and elevate the value of working with marginalized populations [20]. This study also agrees with the findings of a recent qualitative study [46] on experiences with anti-racist teaching in residency programs, including participant suggestions around the need for individual self-reflection of one’s biases and longitudinal integration of anti-racist content across the medical education curriculum [46]. While this study affirms the ongoing need for this training, it also emphasizes the Ufomata et al. [6] observation around the lack of a standard guiding these efforts and the importance of moving towards one, given their nascency and variation in scope and design.

With regard to the latter, accrediting bodies and national licensing examinations play a critical role in setting standards for what content will be taught and evaluated, respectively, and as such should consider deliberating and creating thoughtful standards for content related to race, racism, and racial disparities which draw on the emerging evidence-base, including the results of this study. In pursuing curriculum changes independently, medical schools should ensure they are equipped with the faculty and expertise to teach and apply this knowledge. This may entail dedicating resources to ‘teach the teacher’ on the knowledge base necessary to actively correct the embedded nature of race-based medicine.

To our knowledge, this is the first study to understand and center the perspectives of Black women medical students on race, racism, and anti-racism in medical school curricula. The results of this study equip medical school decision-makers and faculty with a source of actionable feedback for revising and creating content that addresses the need for anti-racist reforms in medical education, grounded in perspectives important to the success of these reforms. The momentum and success of anti-racist reforms may be limited by various roadblocks, including the political environment across levels of government; however, maintaining the status quo promises a health system in which disproportionately sicker populations are perceived as a normal feature of US society. The perspectives of current Black women medical students offer an impetus for change.

### Areas for future research

This study inspires several areas of future inquiry. First, Black women with generational ties in the US were a smaller minority or participants within our study as compared to Black women of first- or second-generation immigrant backgrounds. We believe the former are further underrepresented in the Black medical school enrollment population and physician workforce. Comparative studies of within-group differences in perspectives as they relate to the path to medicine, race, and anti-racism in medical education would advance an understanding of perspectives from persons with marginalized identities. Second, there is a need for rigorous, longitudinal evaluation of existing anti-racist curricular reforms to inform a potential standard for the content, quality, and efficacy of this training.

### Strengths and limitations

Limitations of this study are common across qualitative studies, including a relatively small sample size and potential biases associated with a purposeful sampling approach, including selection bias (e.g., recruiting the most inclined/motivated type of student) and response bias (e.g., ‘social desirability’ or conforming to a perceived expectation of how to respond). Validity measures were implemented to reduce the effects of these limitations. The resulting sample sizes also represented states from across the country and were not concentrated in a singular region; however, the sample is not nationally representative. Further, this study focused on offering actionable insights to inform anti-racist efforts in medical education—a valuable addition to the existing academic medicine literature.

## Supplementary Material

Supplemental Digital Appendix 1 and 2.docx

## Note

1. Anti-racism in this context is defined as an active process of ‘identifying and eliminating racism by changing systems, organizational structures, policies, and practices’ to create a more equitable society [47,48].
