# Supplementary material for: A critical qualitative study to understand current black women medical student perspectives on anti-racist reform in US medical education
Source: Med Educ Online. 2024 Aug 20;29(1):2393436. doi: 10.1080/10872981.2024.2393436 (PMC11340229; doi:10.1080/10872981.2024.2393436)
Supplement: Supplemental Digital Appendix 1 and 2.docx [file ZMEO_A_2393436_SM5625.docx]

## **Supplemental Digital Appendix 1: Semi-structured Interview Questions**

1. Please share with me a little about your journey to medical school?
   1. Follow-up question: What motivated you to this path of becoming a physician?
2. If you will, generally describe the structure of your curriculum (e.g., basic science training in the first two years followed by clinical rotations)?
3. What part of your school’s curriculum teaches concepts surrounding racial disparities and health inequity?
   1. For example, is there content that teaches the concept of ‘structural racism’ i.e., the way in which the historical legacy of slavery and anti-Black racism impacts present-day racial disparities.
4. (If answer to #3 is no). What is your understanding of how racism plays a historical role in health disparities?
   1. Do you perceive there to be a need for content in medical school curriculum on how racism plays a historical role in health disparities?
5. (If answer to #3 is yes). Can you describe the content that is taught around racism and health disparities?
   1. Potential follow-up questions: Is the content currently taught sufficient to counter racist beliefs surrounding the teaching of race as a biological construct? Do you perceive problems or inaccuracies in the way medical school teaches the relationship between race and pathology? If so, please elaborate.
6. The literature points to a need for anti- racist reforms in medical education and training.
   1. What does anti-racism in medical education look like to you? (Anti-racism meaning any action to eliminate the influence of racism in the curriculum, training, environment of future physicians)
   2. Follow-up question: From your perspective, what is needed in the curriculum and training to equip future generations of physicians to understand how racism influences health disparities?
7. What potential issues would you anticipate with implementing anti-racist reforms in medical education at national-scale?
   1. Follow-up questions to be asked to probe on issues that might arise among medical students, medical school faculty and administrators; issues that might arise at a national level?
8. Are there any other thoughts you would like to share about racism in the curriculum and progressing an anti-racist agenda in medical education?

## **Supplemental Digital Appendix 2: Key Theme Participant Quotes Table**

| Domain | Key Theme Description | Select participant quotes |
| --- | --- | --- |
| Critical limitations on course content related to race and racism | **Lack of depth and historical lens related to racism and racial health disparities.** | *“I don't think that I've seen anything specifically talk about slavery and the impact there's always, you know, courses, where we cover the impact of socioeconomic status, but no one is actually talking about why those socioeconomic differences exist in the first place. They're just going to say they exist, and you should keep that into account in your future practices as a doctor… We have also covered the mistrust with African Americans, but not why? No one really goes into a deep dive, and I don't think that my classmates want to be there for things like that. Yeah, that's just reality.”*  *“But if you don't have the historical context, it just seems like we all woke up and somehow Black people aren't healthy in America? And I think redlining, is really important, but I think it's only one facet of why healthcare looks different for our people in this country. So, I think going back to constantly the same parts of like sort of the racial history in this country without focusing on like the wider context as well is a problem that kind of bothered me, but I did appreciate our curriculum.”* |
|  | **Lack of nuanced explanation and exploration of racial inaccuracies, stereotypes, and ongoing misuses of race in medicine.** | *“I'm doing type one diabetes research right now, and I hear so much Black people have been misdiagnosed because they come in and they're just like, oh, you got type 2 diabetes….But I have yet to see in my curriculum – make sure that this person doesn't have type one diabetes. We're still learning that type one diabetes is most prevalent in white people, and you know, it's just like we're feeding these things.*  *And so anyway, I just wish certain things would just [be] said out aloud and directly to students instead of dancing around.”*  *“They were talking about obesity, and they pretty much said like Black people are vulnerable to obesity. And that was it. Nobody explained why that was. We just know they probably obese, that's how that comes off.”*  *“Give us evidence-based resources that say like, even if this is the guidelines, this isn't what this isn't what makes sense anymore. And even if you guys have to memorize this for the boards, here's why you should be questioning that… And critically, thinking about why that's not right, because I feel like not even just for just because that's not great medicine and that's not great science. I feel like that would better prepare us for questioning other guidelines. I feel like that would have been a really great thought exercise and also a critical way of teaching us how to do that in the future when we come across another guideline, I think that's something that's really been missing like they just kind of say, well, that's quite maybe that's not right, but this is what you need to do. And I just feel like they just need to give us the tools to dismantle other structural or other examples of structural racism. And that's not really being given to us at the moment, has the longer we don't have those tools, the longer these systems stay in place.”* |
|  | **Lack of cohesion and continuity of topics introduced.** | *“I couldn't even tell you the last time we had a meeting regarding race because it's just sprinkled here and there. It's sporadic.”*  *“And so if I have the power to create an ideal anti-racist curriculum, it would be treated with the same regard and value as our basic science courses, the same amount of time and course hours and regularity and continuity as anatomy. It would have that continuity. It would have those protected amount of time each week or multiple times a week. I will be treated exactly the same because it's value would be perceived as necessary.”* |
|  | **Lack of actionable guidance and application of knowledge in the context of clinical practice.** | *“You just get all these grim statistics, and you’re like, wow, I’m more likely to die if I have a kid. Like what am I supposed to do with that information? And even not necessarily for myself but like for my patients. What am I supposed to do with that information? So maybe actionable steps instead of just learning these statistics.”* |
|  | **Lack of trained, qualified, and engaged academic instructors on race and racism in medicine.** | *“I would have liked critical theorists involved, people in the humanities who are focused on the anti-racism and anti-Blackness and structural racism and ideas about how to dismantle that, how it came about, people who are experts in that field would be the ones leading these courses in the same way that experts in the field are teaching me cardiology or the cardiovascular system and pulmonology… the courses would be treated and valued as just as would basic sciences science courses are.”*  *“I know that there are thought leaders in these areas that exist on all of these campuses. So, for me, it's kind of frustrating that we have people who are doing research and the work when you know people are called to speak about these issues. These are the people they're pulling from, and they're literally doors away, steps away. Why are we not bringing in these people to provide that education?”* |
|  | **Dissonance between changing curriculum content and lagging change in national board and licensure exams.** | *“As far as national board, that is something that will come up, there will descriptors, and you're immediately supposed to think of whatever problem, right? Just because it says like Black 50-year-old man, so that is something that I've noticed and come up and we've discussed that amongst my classmates, my school doesn't write those. So, like, there's only so much they can do about that.”* |
| Strategies for anti-racist curriculum reform | **Evaluate anti-racist content on the same standard as basic science coursework.** | *“There was an option… I think it would be the only anti-Blackness and racism lecture last year which was done as a pilot for, and it was optional for students, basically no students showed up and the students who did show up were Black.”* |
|  | **Provide exercises that allow students to critically reflect on issues of implicit and explicit bias individually and at a broader level.** | *“I think like reflection is probably a better way…because I think it's more of like personal bias identification and understanding how harmful biases can be. I think that might be more impactful rather than like multiple choice questions on an exam.”*  *“Or even bringing in people that actually are affected by stuff like that and just discussing…I have a friend who has sickle cell and she has to go through loopholes just to get certain pain medications because she fits the demographic that could abuse these medications. You know, she has to fight with physicians just to be treated equally. So someone like that coming to talk about the issues that they face; when we're taught these certain descriptions about certain patients that they should keep an eye out and not default to certain assumptions. I feel something like that would be more would resonate or stick with people more than an implicit bias test.”* |
|  | **Shift away from the traditional lecture format to in-person small-group sessions and discussions.** | *“I would actually prefer if there were in small groups. I feel like it gives people a more comfortable space to actually speak on what is being discussed and for the teacher.”*  *“I think I probably get a little bit more out of small groups than necessarily lecture style because I think questions tend to generate conversations on the topics and that resonates more with me rather than being lectured at.”* |
|  | **Integrate opportunities to interact and engage with marginalized members of the communities within the state of the respective medical school.** | ***“****Definitely engaging with these community partners and getting boots on the ground and seeing those experiences is really key so that you're actually getting exposed to what that experience may be like for folks and also seeing what's needed from the community because it's easy for you to say ‘well, I think people need this’, but then that's not aligned with what the patients actually want.”*  *“…People discuss these issues in the classroom, but then it's then you leave the classroom and never really interact with anybody who's different from you or comes from a different background from you. So, I think maybe just more community engagement.”*  *“I found that when coming here, there just wasn't a lot of experiences for me as an M1 or M2 to really be out in the community like that. So, I feel having that option will help expose whether it's BIPOC students or White peers to the realities of different communities early on in the health care environment.”* |
| Obstacles to scaling anti-racist curriculum reforms at a national-level |  | *“When things are just already established and working for a lot of people and then we want to change and remove things from it, that can be difficult.”*  *“Changes in the curriculum don't necessarily match with the reality of practice sometimes.”*  *“I think it could call in a lot of attention just in this current political environment, that could potentially be very problematic for certain people. And I mean especially with the Supreme Court ruling about affirmative action, I do anticipate there could be like a lot of pushback.”*  *“But if your state is a type of state who you know, they're literally on the news talking about, we can't say the word ‘gay’ in school and then you put this in your medical school curriculum…I don't know how that's going to work out for you.”*  *“Coming from a state that's very liberal, I think we have a lot of leeway to talk about things as freely as we'd like… I would just be nervous to see what more conservative states what would implement in response to that [anti-racist standards]. We're seeing in Florida that they're trying to get rid of African American AP history and so I would just be nervous to see what they teach in some of those other states.”* |
